# World-wide distributions of lactase persistence alleles and the complex effects of recombination and selection

**DOI:** 10.1007/s00439-017-1847-y

**Published:** 2017-10-23

**Authors:** Anke Liebert, Saioa López, Bryony Leigh Jones, Nicolas Montalva, Pascale Gerbault, Winston Lau, Mark G. Thomas, Neil Bradman, Nikolas Maniatis, Dallas M. Swallow

**Affiliations:** 1 Research Department of Genetics, Evolution and Environment, University College London, Darwin Building, Gower Street, London, WC1E 6BT UK; 2 UCL Department of Anthropology, Human Evolutionary Ecology Group, University College London, 14 Taviton Street, London, WC1H 0BW UK; 3 Present Address: Department of Paediatrics, University of Cambridge, Box 116, Level 8, Cambridge Biomedical Campus, Cambridge, CB2 0QQ UK; 4 Present Address: Departmento de Antropología, Facultad de Ciencias Sociales y Jurídicas, Universidad de Tarapacá, 384 Calle Cardenal Caro, Arica, Chile; 5 Present Address: Department of Life Sciences, Faculty of Science and Technology, University of Westminster, 115 New Cavendish Street, London, W1W 6UW UK; 6 Henry Stewart Group, 28/30 Little Russell Street, London, WC1A 2HN UK

## Abstract

The genetic trait of lactase persistence (LP) is associated with at least five independent functional single nucleotide variants in a regulatory region about 14 kb upstream of the lactase gene [−13910*T (rs4988235), −13907*G (rs41525747), −13915*G (rs41380347), −14009*G (rs869051967) and −14010*C (rs145946881)]. These alleles have been inferred to have spread recently and present-day frequencies have been attributed to positive selection for the ability of adult humans to digest lactose without risk of symptoms of lactose intolerance. One of the inferential approaches used to estimate the level of past selection has been to determine the extent of haplotype homozygosity (EHH) of the sequence surrounding the SNP of interest. We report here new data on the frequencies of the known LP alleles in the ‘Old World’ and their haplotype lineages. We examine and confirm EHH of each of the LP alleles in relation to their distinct lineages, but also show marked EHH for one of the older haplotypes that does not carry any of the five LP alleles. The region of EHH of this (B) haplotype exactly coincides with a region of suppressed recombination that is detectable in families as well as in population data, and the results show how such suppression may have exaggerated haplotype-based measures of past selection.

**Electronic supplementary material:**

The online version of this article (doi:10.1007/s00439-017-1847-y) contains supplementary material, which is available to authorized users.

## Introduction

There is now good functional evidence that the genetic trait of persistence of intestinal lactase activity into adult life can be caused by five or more independent single nucleotide variants in a regulatory region (a transcriptional enhancer) upstream of the lactase gene *LCT* (Fang et al. 2012; Ingram et al. 2007; Jensen et al. 2011; Liebert et al. 2016; Olds and Sibley 2003; Troelsen et al. 2003). One of these, −13910*T (rs4988235) (Enattah et al. 2002) has almost reached fixation in some parts of Europe, while others such as −13907*G (rs41525747), −13915*G (rs41380347), −14009*G (rs869051967) and −14010*C (rs145946881) are found at variable frequencies in the Middle East and Africa (Enattah et al. 2008; Ingram et al. 2007, 2009; Tishkoff et al. 2007). Present-day frequencies of these alleles have been attributed to positive selection for lactase persistence, which allows the dietary consumption of animal milk by adult humans without risk of symptoms of lactose intolerance (Allentoft et al. 2015; Bersaglieri et al. 2004; Gallego Romero et al. 2012; Gerbault et al. 2009; Itan et al. 2009; Mathieson et al. 2015; Schlebusch et al. 2013; Sverrisdottir et al. 2014; Tishkoff et al. 2007). The distributions of these alleles have also clearly been influenced by other processes including population expansion, migration and allele surfing, and cultural/environmental processes (reviewed in Gerbault et al. 2011).

Ancient DNA data have the potential to address the question of where and when an allele first occurred at significant frequency. Increased availability of such data for the European lactase persistence (LP) associated allele, −13910*T has allowed some degree of geo-temporal mapping of its distribution, although so far there is insufficient data to track its geographic origin. The earliest occurrences have been reported in Spain, dated to about 5000 years BP (Plantinga et al. 2012), though having been obtained through PCR-based technology, the possibility of contamination cannot be ruled out. Using NGS sequencing, the earliest detections of the allele were in Germany and Sweden about 4000 years BP (Allentoft et al. 2015; Haak et al. 2015; Mathieson et al. 2015) highlighting its very recent expansion (see Supplementary data for full list of references). There are no reports yet of the other alleles in ancient samples, but genetic evidence points to rather recent spread for all five functional variants (Enattah et al. 2008; Jones et al. 2013; Priehodova et al. 2017; Schlebusch et al. 2013; Tishkoff et al. 2007). Estimation of the age of expansion of the European allele using population genetic and modelling approaches places it during the Neolithic period and suggests selection coefficients ranging from 0.8 to 19% (Bersaglieri et al. 2004; Gerbault et al. 2009; Itan et al. 2009). Such coefficients are extraordinarily high in view of the fact that the cultural adaptation of fermentation of milk products, which reduces the lactose concentration, allows milk to be used as a source of calories in the diet of lactase non-persistent people, circumventing its adverse effects (Segurel and Bon 2017).

One inferential approach frequently used to identify signatures of selection is to determine the extended haplotype homozygosity (EHH) of the sequence surrounding a variant of interest (Bersaglieri et al. 2004; Sabeti et al. 2002; Tishkoff et al. 2007). This method is relatively straightforward when only one functional allele is present at appreciable frequency. Such is the case for LP-associated alleles in Europe and Tanzania, i.e. −13910*T (rs4988235) and −14010*C (rs145946881), respectively. However, the occurrence of several different putative selected alleles in the same sample, as is the case in Ethiopia (Jones et al. 2013, 2015), can complicate interpretation, since these alleles can each be associated with different extended haplotypes. Furthermore, Gallego Romero and colleagues (Gallego Romero et al. 2012) recently reported a common extended haplotype that is not associated with LP.

In this study, we evaluate ‘Old World’ allele frequencies of the known functional LP alleles, as well as other alleles within the *LCT* enhancer region, adding extensive new data, examine the haplotype backgrounds of each variant and compare extended haplotype homozygosity with those of the corresponding ancestral haplotypes. We also investigate in detail the level of recombination in the chromosomal region, using the HapMap and Icelandic populations.

## Materials and methods

Samples newly tested for this paper (2056 individuals from 52 populations) included groups collected under the auspices of ethical committee approvals UCLH 99/0196 and 01/0236. DNA was extracted from buccal samples by various adaptations of the phenol chloroform method. Individual samples were grouped according to the country in which they were collected, and the continental geographic region in which the country is located, namely Northwest/Central Europe, South Europe, East/Southeast Europe, the Middle East, West Asia, Central/South Asia and East/Southeast Asia (labelled Europe-N, Europe-S, Europe-E, M-East, Asia-W, Asia-S and Asia-E, respectively, in Table 1, and see Supplementary Table 2a for groupings). A further categorization was made into distinct cultural groups with a minimum sample size of 10 individuals, using self-declared cultural identity/ethnic background, if such information was available, or geographic subgroups within countries where there was more precise information about the sample localization.

**Table 1:** Frequency of enhancer region derived alleles by geographic region for samples newly reported in this paper

Region	*N*	− 14011 C>T*	− 13915 T>G**	− 13910 C>T**	− 13907 C>G**	− 13906 T>A	− 13779 G>C*	− 13744 C>G	− 13730 T>G	− 13603 C>T	− 13495 C>T
Europe-N	744	0.003	–	0.615	–	–	–	–	–	–	0.729
Europe-S	594	0.003	–	0.305	–	–	–	–	0.002	–	0.429
Europe-E	550	0.005	–	0.231	–	–	–	–	–	–	0.388
M- East	892	0.001	0.094	0.041	0.003	–	0.007	0.006	0.004	0.006	0.238
Asia-W	290	–	–	0.094	–	–	–	–	–	–	0.269
Asia-S	562	–	–	0.167	–	0.002	0.005	–	–	–	0.397
Asia-E	480	0.002	–	0.038	–	0.002	–	–	–	–	0.378

All 10 SNPs in the table were found more than once. The SNP −13495 C>T (rs4954490) is outside the enhancer characterised experimentally, but included here because it was sequenced in the same DNA fragment. Of the 12 singletons (Supplementary Table 2a), 6 were novel (−14062 G>A, −14010 G>A, −13964 C>A, −13926 A>C, −13771 A>G, −13693 G>A). Known functional SNPs **; * indicates some evidence for function (Liebert et al. 2016). See Supplementary Table 2a for country groupings

### Enhancer sequencing

*LCT* enhancer sequences from all 2056 DNA samples were obtained from a 706 bp fragment in intron 13 of *MCM6*, PCR amplified as described previously (Ingram et al. 2009; Jones et al. 2013). Supplementary Table 1 shows the primers, locations and cycling conditions. All fragments were sequenced in both directions using a modified version of the Sanger Method and run on an ABI 3730xl DNA Analyzer (Applied Biosystems).

### 80 kb Haplotype background of enhancer variants

For a subset of 880 samples that included 354 of the newly typed samples as well as European, Middle Eastern and African samples, from populations previously analysed by our group (Ingram et al. 2007, 2009; Jones et al. 2013) additional sequencing and genotyping were performed to obtain data to deduce the 80 kb haplotype background of the enhancer variants (see Supplementary Fig. 1 for all variants). Sequences were obtained from two regions flanking the *LCT* enhancer, a 683 bp haplotype-defining region upstream of *LCT* (Hollox et al. 2001) and a 701 bp region in Intron 4 of *MCM6* (Jones et al. 2013). The *LCT* gene region haplotype markers in exon 2 (666 G>A) and exon 17 of *LCT*, (5579 T>C) were genotyped by LGC Genomics, Teddington, Middlesex,UK) using Kompetitive Allele Specific PCR (KASP) technology (http://www.lgcgroup.com/products/kasp-genotyping-chemistry/#.WbAZy62ZM5g).

PHASE v. 2.1.1 (Stephens et al. 2001; Stephens and Donnelly 2003) was used to infer haplotypes for a final data set of 855 individuals (see Supplementary Table 6). Samples with more than 10% missing data as well as positions with alleles occurring only once were excluded from PHASE analysis.

The software, Network (version 4.6.1.1, http://www.fluxus-engineering.com) was used to construct a haplotype network for this 80 kb genetic region.

### Linkage disequilibrium unit (LDU) and genetic (cM) maps

LDU maps (Maniatis et al. 2007) were constructed using data from all the populations of the HapMap Project release #28 (International HapMap3 Consortium 2010). The sex-averaged family cM map based on linkage data from the large Icelandic families was taken from Kong et al. (2010) (sex-averaged.rmap, https://www.decode.com/addendum/).

### Extended haplotype homozygosity (EHH)

In addition to the two *LCT* haplotype markers, a further 34 loci flanking the enhancer were selected for KASP genotyping by LGC Genomics (details above) to extend the haplotype analysis to 1.77 Mb surrounding *LCT*. These SNPs were selected with the aim of distributing them at an average distance of 50 kb apart. This distribution was adjusted to take into account the LDU maps from the Hapmap populations, and in regions of high LD the markers were spread out, while in regions of lower LD they were placed slightly closer. The full set of SNPs is shown in Supplementary Table 4 with their physical positions along the chromosome.

Haplotypes were determined (PHASE v. 2.1.1) for a final set of 837 individuals (of the 855 above) (Supplementary Table 2b) with nearly complete data (samples with > 10% missing were excluded). The full set of SNPs spread across the 1.77 Mb region was used to measure EHH using the Selscan v1.1.0b package (Szpiech and Hernandez 2014), for each major population group (Europe, Africa, Asia and Middle East) and using each of the SNPs under test as core. SNPs with minor allele frequency < 0.05 were not included in the analysis. The integrated haplotype scores (iHS) were also determined using the Selscan v1.1.0b package. We used the physical map as a proxy for the genetic map because when the genetic distance is zero over several SNPs, the iHS algorithm fails to return results for all SNPs.

## Results

Sequencing revealed a total of 22 derived alleles within the *LCT* enhancer region. The ancestral state of these SNPs was determined by sequence comparison with other primate species, and was in each case the same as the common allele in humans. Of the 22 derived alleles, 10 occurred more than once. Table 1 shows their allele frequencies in each major geographic area, apart from Africa, which we have reported previously (Jones et al. 2015). The new data in Table 1 includes three of the five established functional variants (−13910*T, rs4988235; −13907*G, rs41525747 and −13915*G, rs41380347), as well as −14011*T, (rs4988233) and −13779*C (rs527991977) for which there is more limited evidence of function (Liebert et al. 2016). The other two established African functional alleles *(*−14009*G, rs869051967 and 14010*C, rs145946881) were only found as singletons. Our own previously reported African data for this genomic region, as well as data reported in the literature by others, were combined with the new data (Supplementary Table 3, in which references are given) and used to examine the geographic distribution of the five most well established functional variants (Supplementary Fig. 2), and to show the distribution of −13910*T in Europe comparing modern and ancient data (Supplementary Fig. 3).

80 kb haplotypes were determined using PHASE. The numbered haplotypes were also assigned to the previously reported *LCT* gene region haplotypes using the five *LCT* gene region haplotype-defining-SNPs, i.e. −958C>T, −943/2 TC>Del, −678G>A, 666G>A and 5579T>C (Hollox et al. 2001). Supplementary Tables 5 and 6 show the results of the PHASE analysis, and the haplotype backgrounds of the derived alleles for each of the enhancer variants. In agreement with previous studies (Bersaglieri et al. 2004; Coelho et al. 2005; Poulter et al. 2003) nearly all the −13910*T alleles were found to be on the same 80 kb haplotype (**24**) associated with an *LCT* gene region **A** haplotype. Just one −13910*T allele was on a different haplotype in a single UK individual most likely due to a recombination event between (678 A>G) and *LCT* exon 2 (666 G>A), since the haplotype is the same as haplotype **24** up to position − 678. Myles and colleagues (Myles et al. 2005) found 8 similar cases in Moroccan and Algerian Berber populations. Except for three alleles, −13907*G is located on haplotype **21** which is also associated with an **A** haplotype background. −13603*T (haplotype **15**) and most of the −14011*T variants are also associated with the *LCT* haplotype **A**. However, two −14011*T alleles were found associated with different **B** haplotype backgrounds, and if the assignments are correct that might suggest this mutation happened more than once independently, probably in geographically distinct places.

The derived allele −13495*T (rs4954490) located just outside the enhancer region, also occurs as a derived allele on the ancestral **A** haplotype (Supplementary Table 5) and is associated in almost all cases with 13910*T and −13907*G, as well as −14011*T, and −13603*T indicating that −13495*T (rs4954490) predates these enhancer region alleles.

With the combination of loci used in this study, it was possible to distinguish between **B** and **P** haplotypes and confirm that −14010*C lies on a haplotype **82** background, associated exclusively with the **P** haplotype (Jones et al. 2013). Also, in agreement with previous studies (Ingram et al. 2009; Jones et al. 2013), the vast majority of −13915*G alleles are located on a **C**-associated haplotype background (haplotype **56**) and −14009*G mostly on haplotype **26**, associated with the *LCT* haplotype **X**, but just one, with an ancestral **H**, which is also the background of the majority of the −13730*G variants (haplotype **17**). The variants −13806*G and −13779*C exclusively occur on **C**-associated haplotype backgrounds (haplotypes **53** and **52**, respectively). −13913*C resides on the **B** haplotype-associated haplotype **80**.

**Fig. 1:**
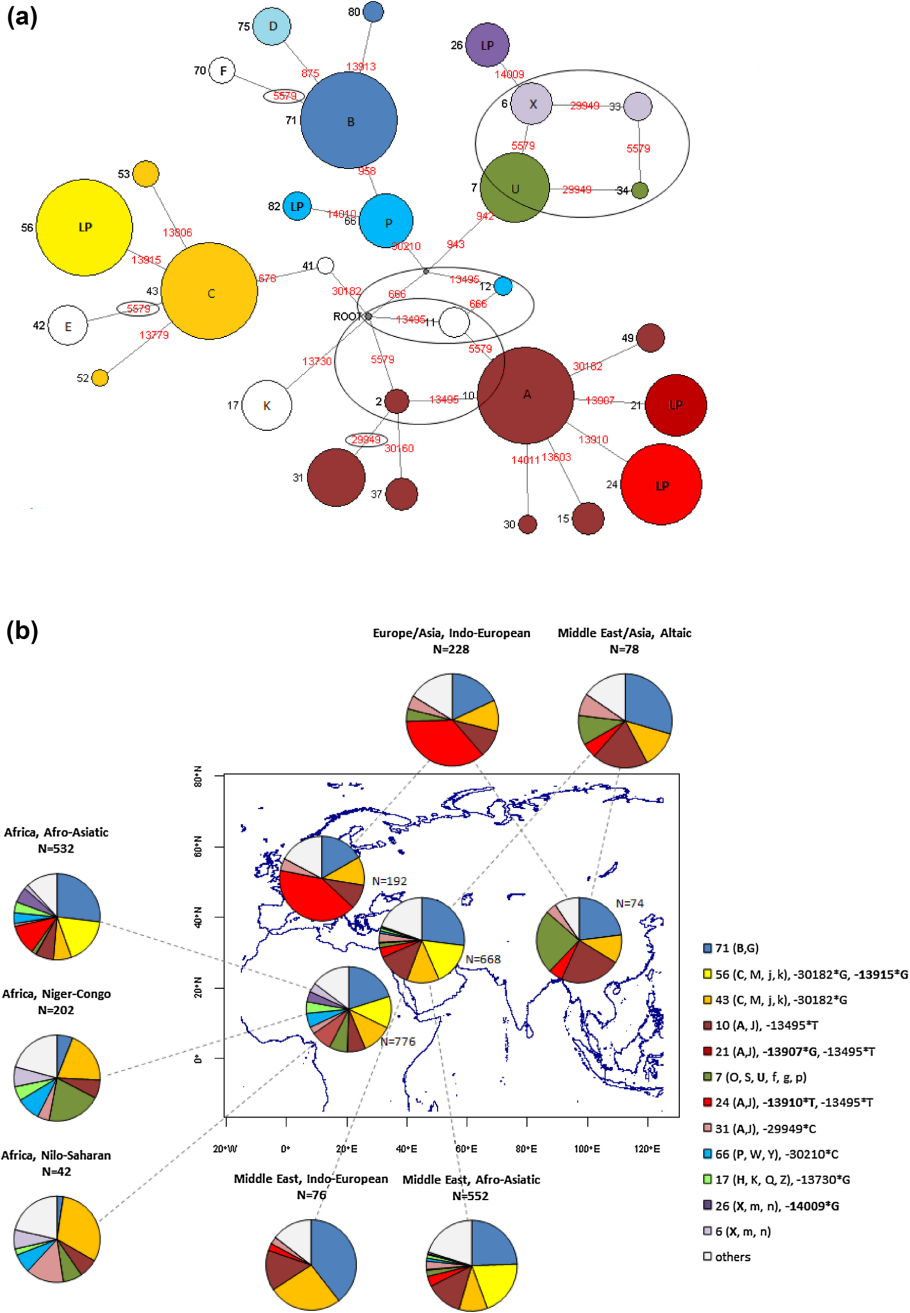
Haplotype network and geographic distribution of haplotypes in Africa, Europe, Middle East and Central Asia. Data from Supplementary Tables 5 and 6. **a** Maximum parsimony neighbour-joining network. The network is made by assuming single stepwise mutational changes, and shows in red the SNPs using their ‘positional’ names (see Supplementary Tables) at each of the mutational steps, and black numbers show the phased haplotypes while black letters show the *LCT* gene region haplotypes of Hollox et al. (2001). Circles are proportional to haplotype count and are coloured with reference to the *LCT* gene region haplotypes (Fig. 1b). Functional alleles are shown as LP. Ovals indicate inferred recombination events where there is more than one appearance of a nucleotide change. Note that branch lengths do not represent evolutionary time scales. **b** Shows the major regional distributions of the phased haplotypes and also shows this subdivided by language group. Derived alleles associated with lactase persistence (LP) are indicated in bold in the key. N represents the number of chromosomes examined per group. The same haplotype colours are used in the Pie segments in **b** as are used in **a**

A haplotype network was constructed for which most branches reflect unique stepwise mutational events, with relatively few recombination events (shown as ovals) needing to be inferred (Fig. 1a). The network illustrates the distant relationships of the five functional variants. The geographic and simplified ethno-linguistic group distribution of these haplotypes is illustrated on a map in Fig. 1b and shown in more detail in Supplementary Table 6.

### Extended haplotype homozygosity (EHH)

**Fig. 2:**
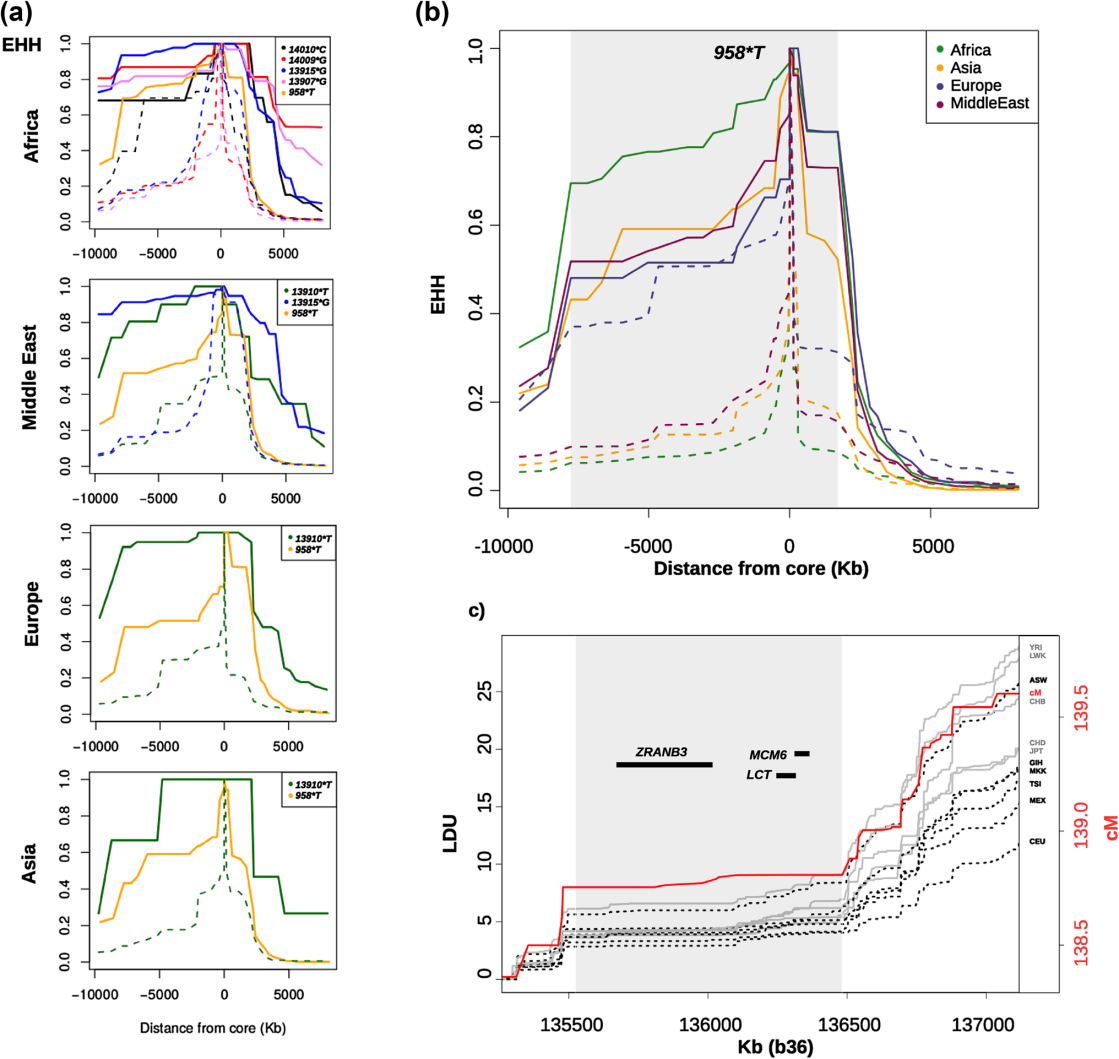
Extended haplotype homozygosity (EHH) in relation to recombination. **a** EHH of the known LP alleles (continuous lines) in comparison with the ancestral A, C, P, X, haplotypes on which they were derived (shown as dashed lines of corresponding colours) and also the B haplotype (using 958*T as core) in the 4 different continental groups. **b** EHH for the B haplotype (958*T—continuous lines) in comparison with all other haplotypes (958*C—dashed lines) in the four continental groups. Note that EHH for 958*T compares all other chromosomes, so that in Europeans the majority of these are −13910*T, and also note that the common ancestral A, C, P, U and X haplotypes fail to show this effect (**a**; Supplementary Fig. 4). **c** The EHH region of the common extended B haplotype (shaded in grey as in **b** is compared with the Linkage Disequilibrium Unit (LDU) maps from the Hapmap populations (black and grey lines; y-axis on the left), see http://www.internationalgenome.org/data for acronyms), and Linkage (cM) maps from the Icelandic families (red line; y-axis on the right). Black dashed lines are populations with LP alleles (> 10%) and grey lines groups in which they are almost absent. Shaded grey area shows the region of the common extended **B** haplotype. Relevant genes from this gene rich region are shown as horizontal lines

EHH analysis was conducted for the four major population groups using each of the functional SNPs as core. All five derived alleles show evidence of EHH. To separate out the effects of the five variants, the chromosomes were separated by major *LCT* haplotype (**A**, **B**, **C**, **P**, **U**, **X**) and the EHH of the derived and ancestral alleles of the same *LCT* background haplotype compared (Fig. 2a). All five functional derived alleles have markedly more extended EHH than the corresponding ancestral haplotypes. The pattern of haplotype decay of the derived alleles is similar in all cases; decaying sharply between 3 and 5 thousand kilobases (kb) on the right of the core SNP and being very much more extended on the left. The *LCT* haplotype **B** shows an EHH pattern somewhat like the LP alleles, and unlike the other ancestral *LCT* haplotypes (Fig. 2a; Supplementary Fig. 4), does show evidence of EHH. Figure 2a, b show the pattern of EHH on the **B** haplotype, using the haplotype-defining SNP, rs56064699 *(*−958*T) as core marker. This haplotype is extended in all four major population groups, although to a lesser extent than the LP-associated allele carrying haplotypes. Strikingly, the decay of EHH occurs at the same position in relation to the markers under test in all four major geographic groups (Fig. 2b).

### EHH in relation to linkage disequilibrium maps (LDU) and centiMorgan (cM)

Since there is little expectation that there has been any selection for the derived allele at rs56064699, because none of the common functional variants occur on this haplotype, and the marker alleles for this haplotype are less frequent in lactose digesters than non-digesters in all groups tested (Ingram et al. 2007; Jones et al. 2013; Ranciaro et al. 2014), we have sought other possible explanations for the apparent high EHH of this haplotype, and examined the pattern of linkage disequilibrium in this region. Figure 2c shows the alignment of the LDU maps constructed for each of the Hapmap populations (acronyms next to the right axis). There is a clear extended region of LD in all populations irrespective of whether LP alleles are present (black dashed lines) or not (grey lines). Since measures of LD not only capture historic recombination, but also are affected by factors such as selection and demography, we sought to examine recombination in family data which only captures recombination events. Notably, the fine-scale lcelandic (deCODE) cM map (red line) coincides with the LDU maps, confirming that the LD pattern is an effect of recombination. Regrettably, there are no suitable data available to construct family cM maps in non-Europeans, but the deCODE map has surprisingly good marker coverage in the region, which means that recombinations can be determined quite accurately, despite the high frequency of LP chromosomes (~ 80%), which decreases the diversity in this population. Moreover (Fig. 2b, c) there is a near correspondence between the extent of the conserved (flat) strong LD/non-recombining region and the most frequent extended **B** haplotype (grey shaded area).

## Discussion

This work provides a comprehensive view of the Old World distribution of known LP-associated alleles; an updated database can be found in Supplementary Table 3. We observe clear geographic distribution differences for each of the derived enhancer region alleles, even though some of them co-occur in East Africa. Although it might be tempting to speculate that the regions of highest frequency are the regions where the alleles originated, simulation modelling (Edmonds et al. 2004; Itan et al. 2009; Klopfstein et al. 2006) has shown that demographic and selection processes can displace spatial allele frequency distributions away from their origin location.

Analysis of the 80 kb haplotype covering the region of *LCT* and the upstream enhancer confirmed a tight association of the LP variants with particular haplotypes, as described previously (Enattah et al. 2008; Ingram et al. 2007, 2009; Tishkoff et al. 2007) and shows that haplotype diversity differs between populations, with the least diversity observed in Northern Europe. With the extension of the haplotype analysis to about 1.8 Mb, it was possible to consider further the putative signatures of selection for the derived alleles associated with LP. EHH analysis shows the haplotypes carrying the derived LP-associated alleles are much longer than their ancestral counterparts, supporting the recent origin of these variants (Sabeti et al. 2002, 2006). Even though the close proximity of the functional alleles does not allow iHS to be measured separately, iHS patterns for the region are consistent with selection in all groups tested (Supplementary Fig. 5).

The *LCT/MCM6* chromosomal region of Europeans had been reported to show one of the strongest ‘signatures’ of selection genome wide (Bersaglieri et al. 2004; Sabeti et al. 2002), namely marked EHH of the derived allele relative to the ancestral allele at rs4988235. While strong selection for LP has been supported by various studies (Aoki 1986; Coelho et al. 2005; Gerbault et al. 2009; Holden and Mace 1997; Itan et al. 2009; Mathieson et al. 2015; Schlebusch et al. 2013; Sverrisdottir et al. 2014), the features of the chromosomal region highlighted here show that other processes, such as recombination, may have influenced the patterns observed.

In particular, we not only confirm the high frequency and wide distribution of the **B** haplotype in this large data set, as also shown in previous studies (Gallego Romero et al. 2012; Hollox et al. 2001; Ingram et al. 2007; Jones et al. 2013), but also further highlight the notable EHH of the **B** haplotype. The **B** haplotype does not carry any known functionally important enhancer alleles subject to positive selection, and is likely to be old, given its widespread geographic distribution; its extended haplotype homozygosity, therefore, requires explanation. We show that the region of EHH overlaps exactly with the region of very little recombination and high LD for all populations, including ones in which no, or very few, LP-associated alleles occur, and consequently cannot have been affected by positive selection for LP. The long gene in the centre of this region of high LD, *ZRANB3* (zinc finger, RNA-binding domain containing 3, a DNA annealing helicase and endonuclease with function for genome stability (UniProtKB, http://www.uniprot.org/), which is important for replication stress response (Weston et al. 2012), is much more likely to have been subject to purifying rather than positive selection.

This lack of recombination inferred from measures of LD in samples from unrelated individuals was confirmed by a corresponding lack of recombination events in the large Icelandic families. Ongoing reduced recombination or suppression of recombination might be attributable to lack of clusters of appropriate sequence motifs required for recombination (Myers et al. 2010) or a structural rearrangement of the chromosome, such as an inversion in this region, in one or more of the haplotypes. This non-recombining block most likely explains the asymmetry of the extended haplotypes carrying the functional SNPs, i.e. the haplotypes extend further downstream of the *LCT* gene even though the functional SNPs (under selection) are located within *MCM6* upstream of *LCT.* This asymmetry can be seen but was not commented on in previous work (Bersaglieri et al. 2004). One could also speculate that a chromosomal rearrangement(s) might have assisted in driving the causative alleles to higher frequency, by transmission distortion similar to that found in other studies (Didion et al. 2016; Odenthal-Hesse et al. 2014). This might contribute to a more rapid increase in frequency and help to explain why the effect of selection seems so high for a phenotype whose selective advantage(s) are still somewhat elusive and environmentally variable (reviewed in Segurel and Bon 2017).

More broadly, our results indicate that regions of the genome in which there has been restricted recombination and where there are relatively few common haplotypes world-wide can give inflated EHH and iHS results, and thus possibly misleading interpretations as to the real extent of selection, when using haplotype-based measures.

## Electronic supplementary material

Below is the link to the electronic supplementary material.

Supplementary material 1 (PDF 1937 kb)

Supplementary material 2 (XLS 135 kb)
