# Supplementary material for: World-wide distributions of lactase persistence alleles and the complex effects of recombination and selection
Source: Hum Genet. 2017 Oct 23;136(11):1445–53. doi: 10.1007/s00439-017-1847-y (PMC5702378; doi:10.1007/s00439-017-1847-y)
Supplement: Supplementary file 1 — Supplementary material 1 (PDF 1937 kb) [file 439_2017_1847_MOESM1_ESM.pdf]

Online resource

World-wide distributions of lactase persistence alleles and the complex effects of recombination and selection

Anke Liebert, Saioa López, Bryony Leigh Jones, Nicolas Montalva, Pascale Gerbault, Winston Lau, Mark G. Thomas, Neil Bradman, Nikolas Maniatis and Dallas M Swallow.

Tables

Supplementary Table 1: Primers and conditions used for PCR amplification and sequencing. See supplementary Figure 1 for locations

Genetic region	Location (bp 5' of *LCT* start of transcription)	Primer name	Primer name in previous publications	Primer sequence (5'-3')	Product length	Annealing temperature (°C)	cycles
LCT enhancer:<br>*MCM6*, intron 13	-14,163	MCM6i13<br>MCM6778	MCM6i13 (Ingram et al 2007)<br>MCM6778 (Ingram et al 2009)	GGACATACTAGAATTCACTGCAAATAC<br>CCTGTGGGATAAAAGTAGTGATTG	706	58	38
MCM6, intron 4	-30,461	MCM6_intron4f<br>MCM6_intron4r	Control region 1 (f) (Jones et al 2013)<br>Control region 1 (r) (Jones et al 2013)	ACCCTCAGATTTCAGCAGGAC<br>ACTCCATGATGATTCAAGCAGC	683	58	39
LCT, hapdef region	-1,162	LCT_far_prom_f<br>LCT_far_prom_r	Control region 2 (f) (Jones et al 2013)	ATCCACATTCTACAGGTGACAA<br>GACCAACACAAAAACCTCAGAC	701	59	38

Supplementary Tables 2 and 3: See Supplementary Data tables.xls.

Database of derived functional alleles, Table 2a showing new data, Table 2b listing samples studied for EHH and Table 3 showing a revised Database of 'Old World' LP allele frequency data.

Supplementary Table 4: Variants included in EHH analysis:

a) Chromosomal location and allelic information for variants detected by sequencing of the DNA fragment covering the enhancer. Coordinates are shown for Human Genome build 38 and 36.
b) Chromosomal location and allelic information for the 6 SNPs typed by sequencing of the two DNA fragments flanking the enhancer fragment. Coordinates are shown for Human Genome build 38 and 36.
c) Chromosomal location and allelic information for the 36 flanking SNPs typed by KASP for EHH. Distance in kb from the preceding marker is shown, together with the minor allele frequencies of the 1000 genomes data for CEU, YRI and CHB. Coordinates are shown for Human Genome build 36.

a)

No.	rs ID	SNP	b38 (Kb)	b36 (Kb)	SNP (Chr +)
1	rs4954490	-13495 C>T	135,850,661	136,324,701	G>A
2	rs56348046	-13603 C>T	135,850,769	136,324,809	G>A
3	rs4954492	-13730 T>G	135,850,896	136,324,936	T>C
4		-13744 C>G	135,850,910	136,324,950	G>C
5	rs527991977	-13779 G>C	135,850,965	136,325,005	C>G
6	ss820496565	-13806 A>G	135,850,972	136,325,012	T>C
7	rs41525747	-13907 C>G	135,851,073	136,325,113	G>C
8	rs4988235	-13910 C>T	135,851,076	136,325,116	G>A
9	rs41456145	-13913 T>C	135,851,079	136,325,119	A>G
10	rs41380347	-13915 T>G	135,851,081	136,325,121	A>C
11	rs869051967	-14009 T>G	135,851,175	136,325,215	A>C
12	rs145946881	-14010 G>C	135,851,176	136,325,216	C>G
13	rs4988233	-14011 C>T	135,851,177	136,325,217	G>A

b)

No.	rs ID	SNP	b38 (Kb)	b36 (Kb)	SNP (Chr+)
1	**rs56211644**	**-678 A>G****	135,837,856	136,311,896	T>C
2	**rs78205226**	**-875 G>A**	135,838,053	136,312,093	C>T
3	**rs56064699**	**-958 C>T ***	135,838,136	136,312,176	G>A
4	rs4988172	-29949 G>C	135,867,116	136,341,156	C>G
5	rs56263017	-30182 A>G	135,867,349	136,341,389	T>C
6	rs1435577	-30210 G>C	135,867,377	136,341,417	C>G

*Note that this SNP is called T-956 by (Ranciaro et al. 2014) and although reported to be associated with LP in Afroasiatic language speaking Kenyans, it is in fact the lack of LP that is significantly associated with the T allele.
**Note also that G-676 in Ranciaro et al is the same as -678A >G. The two nucleotide difference is attributable to the presence of a discrepancy of two nucleotides in a run of 22 Ts at positon 135837614-135837635 in the genome browser build 38, while 24 were found in our sequences.

c)

No.	SNP	Position on Chromosome b36 (Kb)	Distance from upstream SNP (Kb)	SNP (Chr +)	Ancestral allele	MAF 1000 genomes	MAF in HapMap pop. CEU	YRI	CHB
1	rs1446525	135354.317		A/G	A	G = 0.288/629	0.60	0.16	0.07
2	rs4954209	135454.378	100	G/T	T	T = 0.348/761	0.18	0.37	0.62
3	rs2874739	135535.377	81	C/T	T	T = 0.347/757	0.12	0.75	0.20
4	rs1869829	135594.032	59	A/G	G	A = 0.385/840	0.81	0.02	0.38
5	rs2305248	135644.782	51	A/G	G	G = 0.343/748	0.12	0.74	0.20
6	rs1900741	135718.970	74	C/T	T	C = 0.431/941	0.91	0.25	0.14
7	rs1561277	135808.531	90	A/C	A	C = 0.264/577	0.74	1.00	1.00
8	rs9798267	135846.261	38	A/G	A	G = 0.277/604	0.08	0.66	0.15
9	rs6709132	135949.042	103	A/G	G	G = 0.211/460	0.08	0.31	0.15
10	rs3806502	136004.743	56	C/T	T	T = 0.327/714	0.12	0.76	0.15
11	rs4954265	136040.695	36	A/G	G	G = 0.270/590	0.06	0.67	0.15
12	rs961360	136110.128	69	A/G	A	G = 0.315/687	0.08	0.30	0.43
13	rs4954278	136124.761	15	C/T	C	T = 0.181/396	0.08	0.38	0.15
14	rs6430585	136223.397	99	A/C	C	A = 0.292/637	0.14	0.30	0.22
15	rs10188066	136255.983	33	A/G	G	G = 0.455/993	0.17	0.79	0.34
16	**rs2278544***	136262.580	7	A/G	A	G = 0.492/1074	0.82	0.21	0.40
17	rs2304370	136278.205	16	A/G	G	A = 0.254/555	0.11	0.38	0.20
18	**rs3754689***	136307.216	29	C/T	C	T = 0.339/740	0.09	0.49	0.41
19	rs182549	136333.224	26	C/T	C	T = 0.234/510	-	-	-
20	rs309152	136373.722	40	A/G	G	G = 0.321/702	0.08	0.44	0.42
21	rs6430594	136435.643	62	A/G	A	G = 0.198/432	0.09	0.09	0.16
22	rs309137	136482.421	47	C/T	C	T = 0.376/821	0.79	0.02	0.35
23	rs2090660	136535.189	53	C/T	C	T = 0.269/588	0.20	0.11	0.23
24	rs6430600	136552.835	18	A/G	A	A = 0.343/749	0.29	0.62	0.19
25	rs12691874	136596.944	44	A/G	G	A = 0.339/740	0.60	0.10	0.19
26	rs953387	136623.640	27	A/C	A	T = 0.460/1004	0.71	0.56	0.16
27	rs1016269	136657.132	33	A/G	G	A = 0.279/610	0.17	0.24	0.38
28	rs7371043	136693.406	36	C/T	T	T = 0.158/344	0.08	0.18	0.30
29	rs4074120	136743.057	50	C/T	T	C = 0.371/810	0.20	0.28	0.51
30	rs12465599	136791.320	48	A/G	G	G = 0.439/958	0.61	0.55	0.27
31	rs6715450	136838.201	47	A/G	A	A = 0.346/756	0.27	0.35	0.40
32	rs543721	136878.027	40	G/T	G	T = 0.411/897	0.37	0.26	0.58
33	rs12618749	136921.944	44	C/T	C	T = 0.228/497	0.09	0.20	0.41
34	rs16834591	136979.740	58	A/G	A	A = 0.260/567	0.09	0.31	0.37
35	rs580879	137030.609	51	C/T	T	T = 0.257/562	0.19	0.53	0.14
36	rs6711718	137123.482	93	C/T	T	C = 0.451/985	0.50	0.48	0.20

*Note that rs2278544 is 5579C>T and rs3754689 is 666G>A

Supplementary Table 5: *LCT* enhancer/ promotor and gene haplotypes inferred by PHASE. Low frequency SNPs and haplotypes (<5) are not shown. Previous haplotype designations shown in bold are the most probable equivalents (Hollox et al. 2001). N: number of chromosomes. The colour scheme is consistent with the pie charts in Fig 1b and network diagram in Fig1a. Haplotype defining SNPs shown in bold.

**Haplotype ID**	-30210 G>C	-30182 A>G	-30160 A>T	-29949 G>C	-14011 C>T	-14010 G>C	-14009 T>G	-13915 T>G	-13913 T>C	-13910 C>T	-13907 C>G	-13806 A>G	-13779 G>C	-13730 T>G	-13603 C>T	-13495 C>T	**-958 C>T**	**-943/42 TC>Del>TG** (1 TC, 2 DEL)	**-875 G>A**	**-678 A>G**	**666 G>A**	**5579 T>C**	**N**	**Haplotypes**
71	C	.	.	.	.	.	.	.	.	.	.	.	.	.	.	.	T	.	.	.	A	.	388	**B**, G
56	.	G	.	.	.	.	.	G	.	.	.	.	.	.	.	.	.	.	.	G	.	.	204	**C**, M, j, k
43	.	G	.	.	.	.	.	.	.	.	.	.	.	.	.	.	.	.	.	G	.	.	200	**C**, M, j, k
10	.	.	.	.	.	.	.	.	.	.	.	.	.	.	.	T	.	.	.	.	.	C	173	**A**, J
24	.	.	.	.	.	.	.	.	.	T	.	.	.	.	.	T	.	.	.	.	.	C	110	**A**, J
7	.	.	.	.	.	.	.	.	.	.	.	.	.	.	.	.	.	2	.	.	A	.	83	O, S, **U**, f, g, p
21	.	.	.	.	.	.	.	.	.	.	G	.	.	.	.	T	.	.	.	.	.	C	63	**A**, J
31	.	.	.	C	.	.	.	.	.	.	.	.	.	.	.	.	.	.	.	.	.	C	56	**A**, J
66	C	.	.	.	.	.	.	.	.	.	.	.	.	.	.	.	.	.	.	.	A	.	48	**P**, W, Y
17	.	.	.	.	.	.	.	.	.	.	.	.	.	G	.	.	.	.	.	.	.	.	41	H, K, Q, Z
26	.	.	.	.	.	.	G	.	.	.	.	.	.	.	.	.	.	2	.	.	A	C	33	**X**, m, n
6	.	.	.	.	.	.	.	.	.	.	.	.	.	.	.	.	.	2	.	.	A	C	30	**X**, m, n
42	.	G	.	.	.	.	.	.	.	.	.	.	.	.	.	.	.	.	.	G	.	C	27	E
75	C	.	.	.	.	.	.	.	.	.	.	.	.	.	.	.	T	.	A	.	A	.	26	D
15	.	.	.	.	.	.	.	.	.	.	.	.	.	.	T	T	.	.	.	.	.	C	19	**A**, J
37	.	.	T	.	.	.	.	.	.	.	.	.	.	.	.	.	.	.	.	.	.	C	19	**A**, J
11	.	.	.	.	.	.	.	.	.	.	.	.	.	.	.	T	.	.	.	.	.	.	17	H, K, Q, Z
82	C	.	.	.	.	C	.	.	.	.	.	.	.	.	.	.	.	.	.	.	A	.	14	**P**, W, Y
49	.	G	.	.	.	.	.	.	.	.	.	.	.	.	.	T	.	.	.	.	.	C	14	**A**, J
33	.	.	.	C	.	.	.	.	.	.	.	.	.	.	.	.	.	2	.	.	A	C	14	**X**, m, n
70	C	.	.	.	.	.	.	.	.	.	.	.	.	.	.	.	T	.	.	.	A	C	13	F, **I**
53	.	G	.	.	.	.	.	.	.	.	.	G	.	.	.	.	.	.	.	G	.	.	12	**C**, M, j, k
2	.	.	.	.	.	.	.	.	.	.	.	.	.	.	.	.	.	.	.	.	.	C	10	**A**, J
80	C	.	.	.	.	.	.	.	C	.	.	.	.	.	.	.	T	.	.	.	A	.	8	**B**, G
12	.	.	.	.	.	.	.	.	.	.	.	.	.	.	.	T	.	.	.	.	A	.	6	**P**, W, Y
30	.	.	.	.	T	.	.	.	.	.	.	.	.	.	.	T	.	.	.	.	.	C	5	**A**, J
52	.	G	.	.	.	.	.	.	.	.	.	.	C	.	.	.	.	.	.	G	.	.	5	**C**, M, j, k
34	.	.	.	C	.	.	.	.	.	.	.	.	.	.	.	.	.	2	.	.	A	.	5	O, S, **U**, f, g, p
41	.	G	.	.	.	.	.	.	.	.	.	.	.	.	.	.	.	.	.	.	.	.	5	H, K, Q, Z

Supplementary Table 6: Distribution of the most common haplotypes across 28 populations studied. Abbreviations for language families: NC: Niger-Congo, AA: Afro-Asiatic, NS: Nilo-Saharan, A: Altaic, IE: Indo-European. HT: Haplotype, Core: *LCT* haplotype, N: Number of chromosomes.

				**HT**	**71**	**56**	**43**	**10**	**24**	**7**	**21**	**31**	**66**	**17**	**26**	**6**
					(**B**,G)	(**C**, M, j, k)<br>-30182*G<br>-13915*G	(**C**, M, j, k)<br>-30182*G	(**A**, J)<br>-13495*T	(**A**,J)<br>-13910*T<br>-13495*T	(O, S, **U**, f, g, p)	(**A**,J)<br>-13907*G<br>-13495*T	(**A**,J)<br>-29949*C	(**P**, W, Y)<br>-30210*C	(**H**, K, Q, Z)<br>-13730*G	(**X**, m, n)<br>-14009*G	(**X**, m, n)
**Region**	**Country**	**Populations**	**Lang family**	**N**												
Africa	Cameroon	Mambila	NC	40	-	-	0.200	0.075	-	0.325	-	0.025	0.050	0.075	-	0.150
	Ethiopia	Afar	AA	124	0.274	0.185	0.024	0.065	-	0.024	0.242	-	0.032	0.032	0.008	0.008
		Amhara	AA	80	0.375	0.050	0.138	0.125	-	0.013	0.025	-	0.025	0.050	0.038	0.013
		Oromo	AA	124	0.403	0.145	0.024	0.065	-	0.032	0.048	0.024	0.032	0.024	0.024	0.032
		Shabo	NS	42	0.024	-	0.310	0.071	-	0.071	-	0.143	0.071	0.024	-	0.071
	Ghana	Asante	NC	40	0.100	-	0.175	0.075	-	0.175	-	0.075	0.075	0.075	-	0.050
	Malawi	Chewa	NC	40	0.025	-	0.325	0.100	-	0.125	-	0.050	0.100	0.025	-	0.075
	Sudan	BeniAmer	AA	128	0.125	0.266	0.078	0.047	-	-	0.172	0.008	0.063	0.039	0.133	0.008
		Jaali	AA	76	0.171	0.197	0.118	0.053	0.013	0.013	0.013	0.026	0.039	0.079	0.092	0.026
	Tanzania	Chagga	NC	82	0.085	-	0.146	0.049	-	0.195	-	0.037	0.110	0.049	-	0.049
Central	Mongolia	Khalka	A	38	0.211	-	0.105	0.342	0.026	0.211	-	0.026	-	-	-	-
Asia	Nepal	Tharu	IE	36	0.250	-	0.111	0.111	0.083	0.278	-	0.056	-	-	-	-
Europe	UK	Northern European	IE	40	0.075	-	0.075	0.050	0.650	-	-	0.025	-	-	-	-
	Italy	..	IE	32	0.219	-	0.156	0.219	0.031	-	-	0.063	-	-	-	-
	Norway	..	IE	40	0.025	-	0.100	0.025	0.850	-	-	-	-	-	-	-
	Romania	..	IE	40	0.225	-	0.150	0.050	0.150	-	-	0.025	-	-	-	-
	Ukraine	..	IE	40	0.300	-	0.075	0.150	0.300	-	-	0.125	-	-	-	-
Middle	Iran	Iranians	IE	76	0.395	-	0.263	0.145	0.026	-	-	0.026	-	-	-	-
East	Israel	Israeli Arabs	AA	40	0.325	0.025	0.100	0.175	0.025	-	-	0.050	0.025	0.025	0.025	-
		Israeli Bedouin	AA	32	0.250	0.188	0.125	0.125	0.031	0.031	-	0.063	0.031	0.031	-	-
		Palestinians	AA	38	0.316	0.026	0.105	0.158	0.026	0.026	-	0.105	0.026	-	-	-
	Jordan	Jordanian Bedouin	AA	44	0.250	0.386	0.023	0.068	0.068	-	-	-	-	-	-	-
	Kuwait	Kuwaiti	AA	56	0.268	0.268	0.161	0.054	0.036	0.036	-	-	-	0.018	0.018	-
	Saudi Arabia	Saudi Bedouin	AA	40	0.150	0.425	0.050	0.100	-	0.025	-	-	0.050	0.025	-	0.025
	Syria	Syrians	AA	82	0.390	0.037	0.122	0.232	-	-	-	0.024	-	-	-	-
	Turkey	AnatolianTurks	A	40	0.375	-	0.150	0.050	0.075	-	-	0.125	-	-	-	-
	Yemen	Yemeni Hadramaut	AA	154	0.182	0.234	0.117	0.136	0.019	0.045	0.013	0.013	0.006	0.006	-	0.006
		Yemeni Sena	AA	66	0.152	0.212	0.061	0.076	0.136	-	-	0.061	-	0.030	-	0.015

Figures

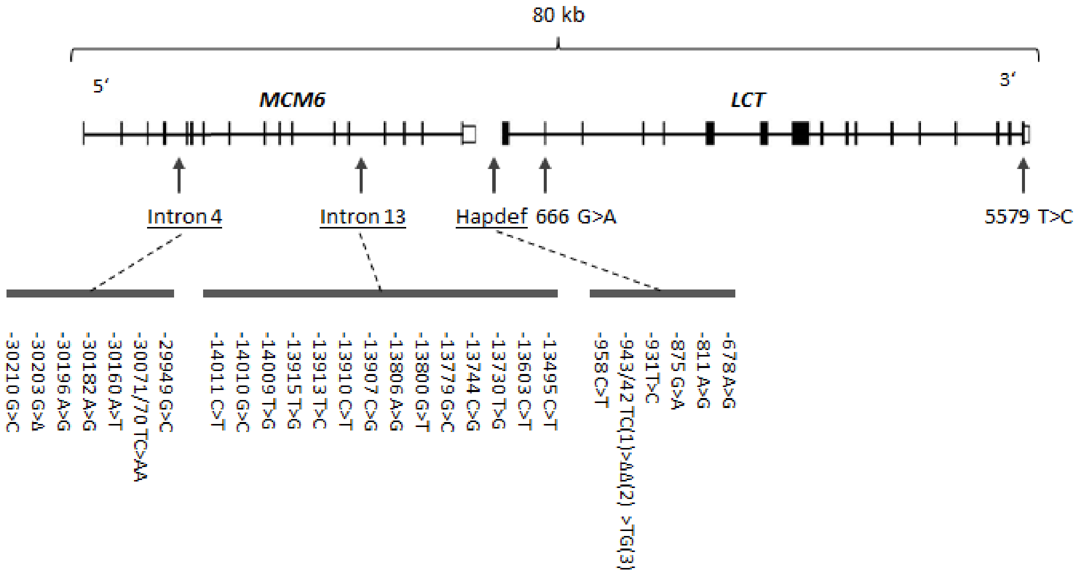

Supplementary Figure 1: All variants included in haplotype analysis of the 80 kb region and their positions in relation to *LCT* and *MCM6*

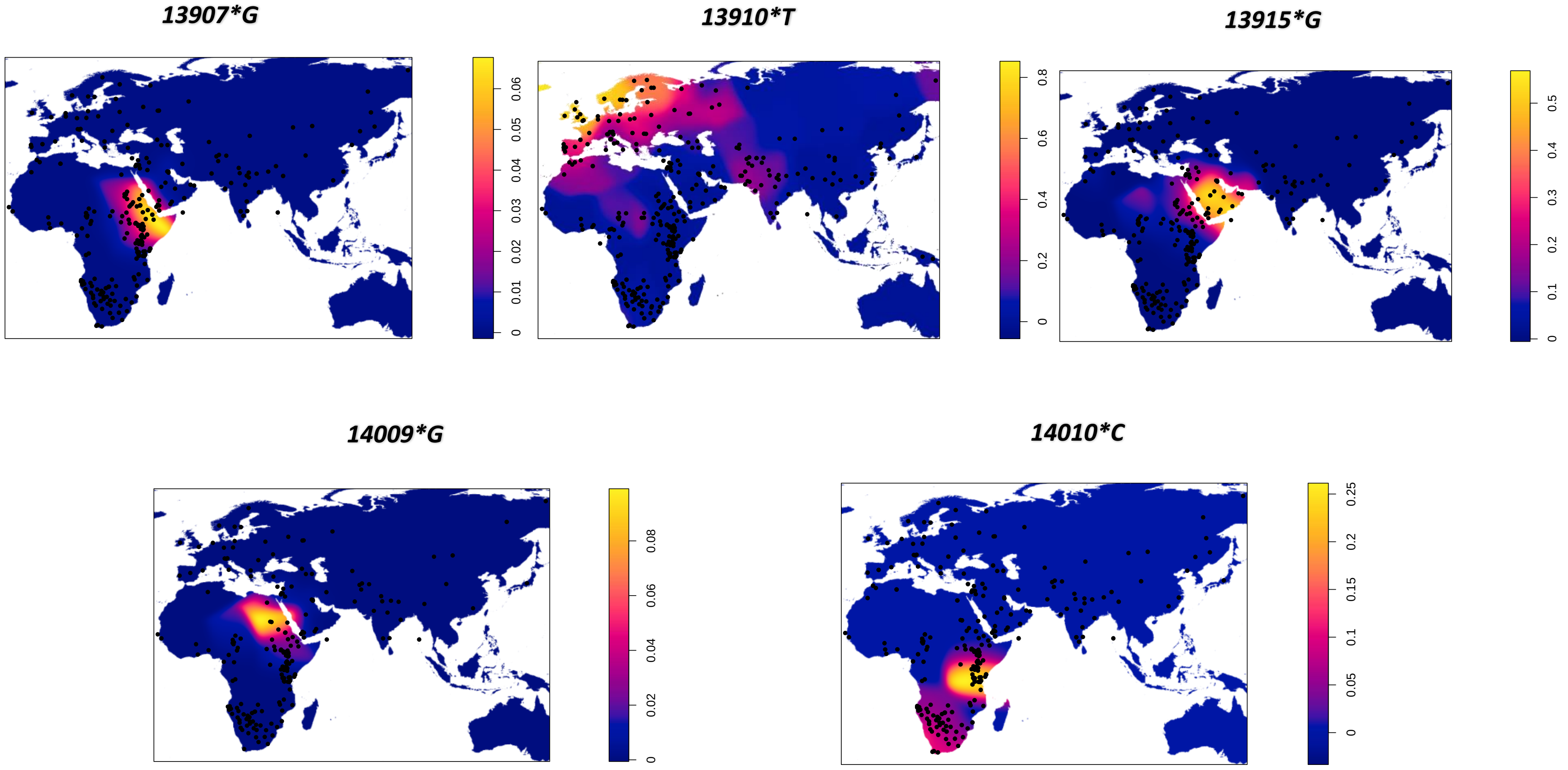

Supplementary Figure 2: Geographic distributions of *LP* causative variants *-13910*T, -13915*G, -13907*G, -14009*G, -14010*C* in the old world. Contour maps were constructed by kernel density estimation as implemented in ‘R’ (R version 3.3.2 (2016-10-31) -- "Sincere Pumpkin Patch" on Mac-OS X Sierra10.12.6) using the *spatstat* package (Baddeley and Turner 2005) and included weighting for sample size. Interpolation smoothing was conducted at the lowest value of sigma (SD=4.0) allowable for all of the variants from the heterogeneous data available.

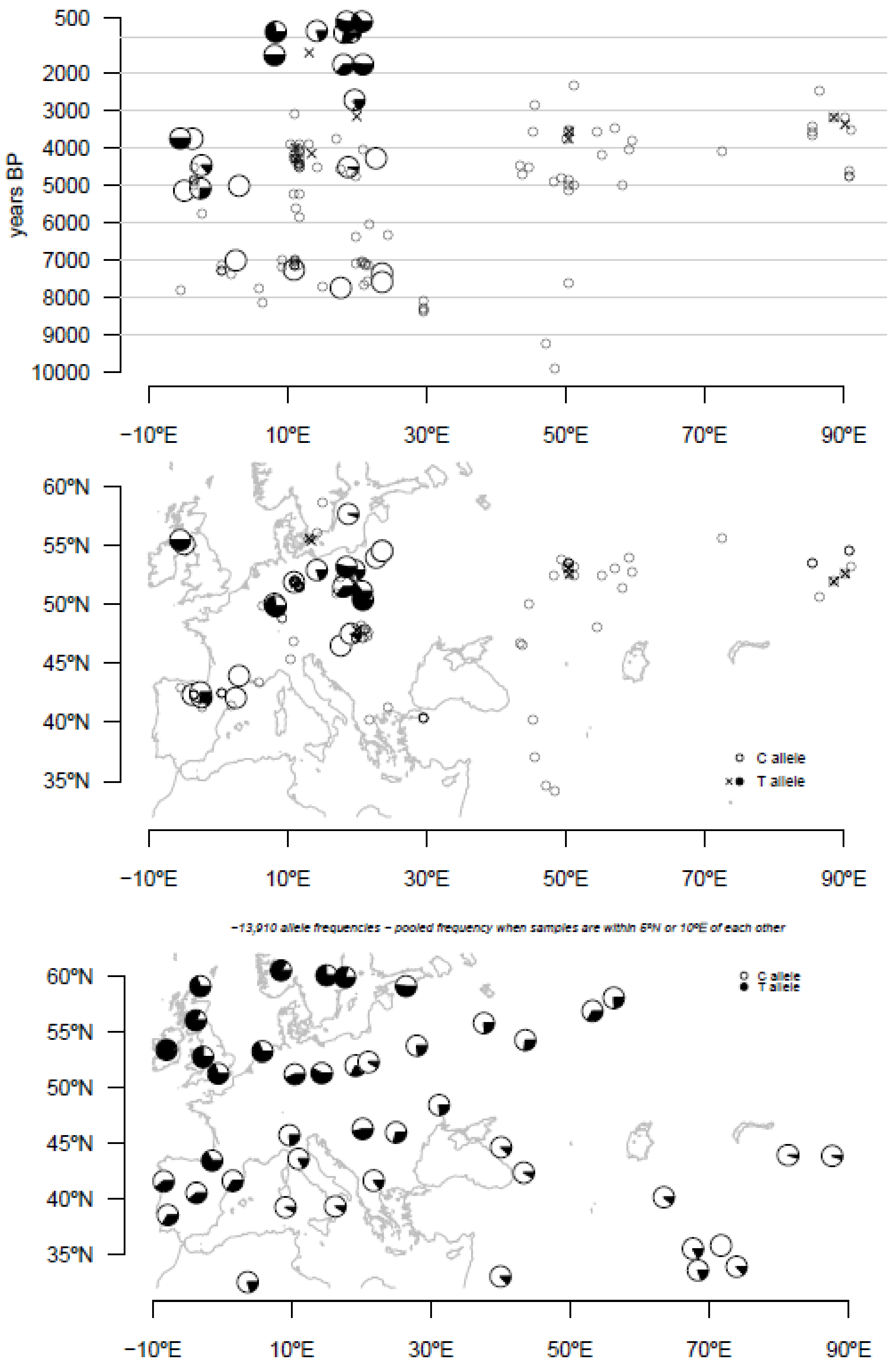

Supplementary Figure 3: Ancient DNA data for *-13910*T* (rs4988235) and comparison with modern data.
Large circles are Pie charts of PCR data and black corresponds to T allele; small symbols NGS data with black crosses for the T allele. The first and second charts show the same data plotted by a) date and b) geography. Data taken from (Allentoft et al. 2015; Broushaki et al. 2016; Burger et al. 2007; Cassidy et al. 2016; Gamba et al. 2014; Haak et al. 2015; Keller et al. 2012; Lacan et al. 2011; Lazaridis et al. 2014; Malmstrom et al. 2009; Mathieson et al. 2015; Nagy et al. 2011; Olalde et al. 2014; Olalde et al. 2015; Plantinga et al. 2012; Ploszaj et al. 2015; Sverrisdottir et al. 2014; Witas et al. 2015) . The third chart shows the same modern data as is interpolated in Supplementary Figure 2.

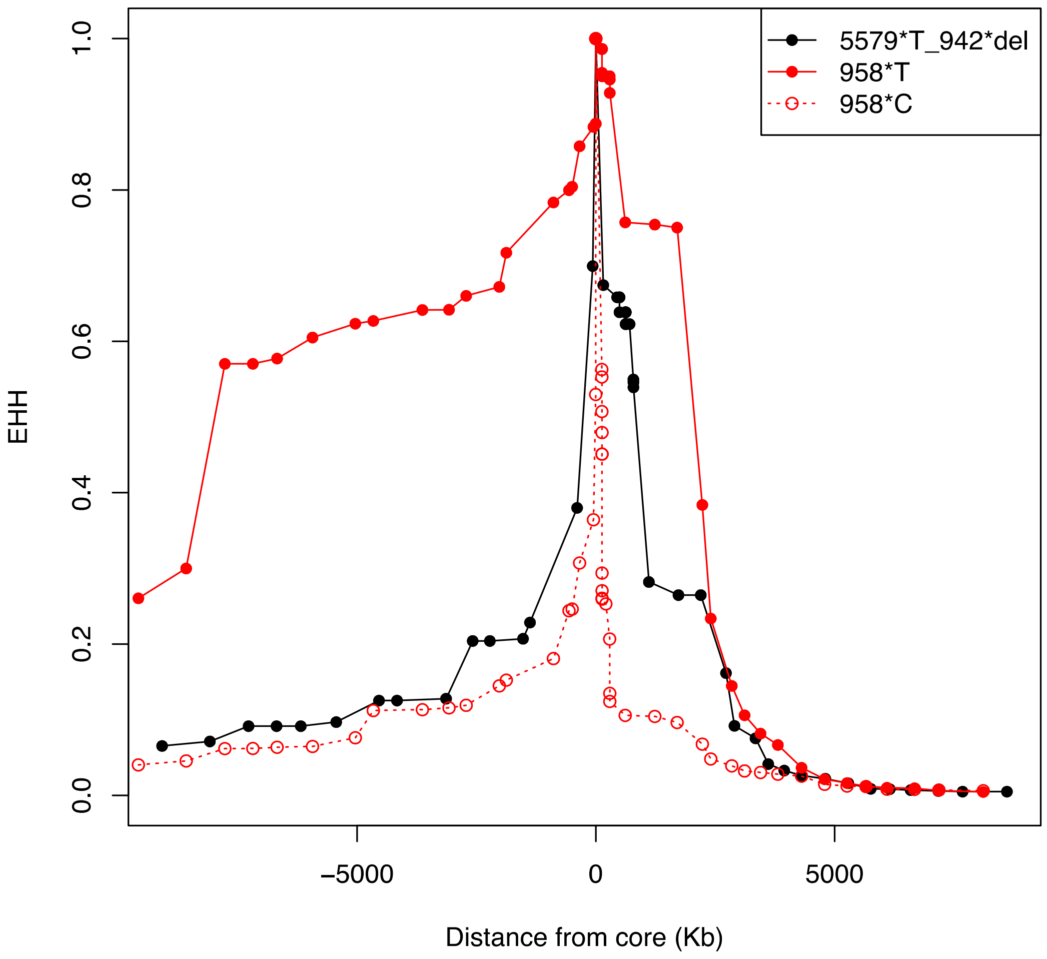

Supplementary Figure 4: EHH of the U and B haplotypes using world-wide data. -5579*T and 942*del are the key SNPs that define the **U** haplotype. -958*T defines the **B** haplotype while -958*C is all other haplotypes.

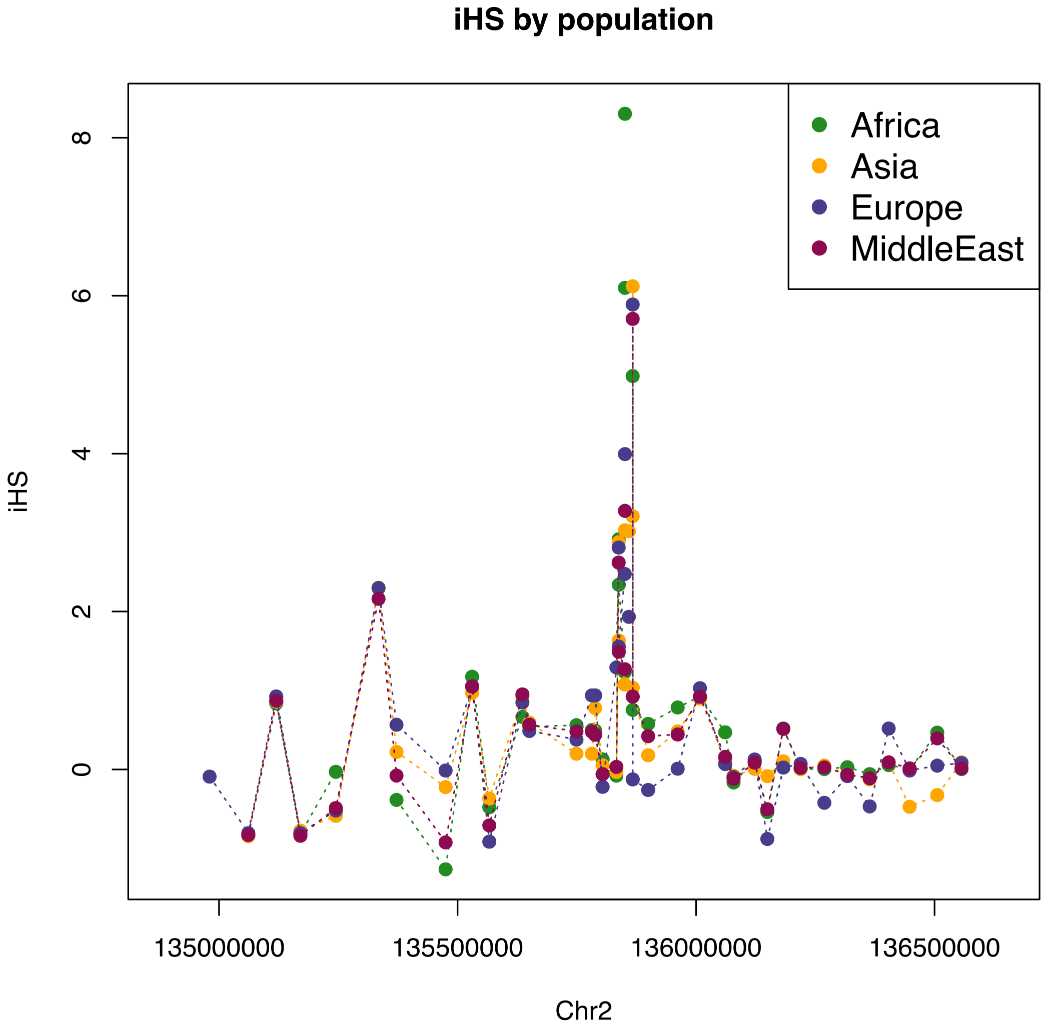

Supplementary Figure 5: iHS as a measure of selection, calculated by population. A positive signal of selection is seen for all four groups (Build 36).

References

Allentoft ME, Sikora M, Sjogren KG, Rasmussen S, Rasmussen M, Stenderup J*, et al.* (2015) Population genomics of Bronze Age Eurasia. Nature 522: 167-72. doi: 10.1038/nature14507

Baddeley B, Turner R (2005) Spatstat: an R package for analyzing spatial point patterns. J Stat Softw 12: 1-42.

Broushaki F, Thomas MG, Link V, Lopez S, van Dorp L, Kirsanow K*, et al.* (2016) Early Neolithic genomes from the eastern Fertile Crescent. Science 353: 499-503. doi: 10.1126/science.aaf7943

Burger J, Kirchner M, Bramanti B, Haak W, Thomas MG (2007) Absence of the lactase-persistence-associated allele in early Neolithic Europeans. Proc Natl Acad Sci U S A 104: 3736-41. doi: 10.1073/pnas.0607187104

Cassidy LM, Martiniano R, Murphy EM, Teasdale MD, Mallory J, Hartwell B*, et al.* (2016) Neolithic and Bronze Age migration to Ireland and establishment of the insular Atlantic genome. Proc Natl Acad Sci U S A 113: 368-73. doi: 10.1073/pnas.1518445113

Gamba C, Jones ER, Teasdale MD, McLaughlin RL, Gonzalez-Fortes G, Mattiangeli V*, et al.* (2014) Genome flux and stasis in a five millennium transect of European prehistory. Nat Commun 5: 5257. doi: 10.1038/ncomms6257

Haak W, Lazaridis I, Patterson N, Rohland N, Mallick S, Llamas B*, et al.* (2015) Massive migration from the steppe was a source for Indo-European languages in Europe. Nature 522: 207-11. doi: 10.1038/nature14317

Keller A, Graefen A, Ball M, Matzas M, Boisguerin V, Maixner F*, et al.* (2012) New insights into the Tyrolean Iceman's origin and phenotype as inferred by whole-genome sequencing. Nat Commun 3: 698. doi: 10.1038/ncomms1701

Lacan M, Keyser C, Ricaut FX, Brucato N, Duranthon F, Guilaine J*, et al.* (2011) Ancient DNA reveals male diffusion through the Neolithic Mediterranean route. Proc Natl Acad Sci U S A 108: 9788-91. doi: 10.1073/pnas.1100723108

Lazaridis I, Patterson N, Mittnik A, Renaud G, Mallick S, Kirsanow K*, et al.* (2014) Ancient human genomes suggest three ancestral populations for present-day Europeans. Nature 513: 409-13. doi: 10.1038/nature13673

Malmstrom H, Gilbert MT, Thomas MG, Brandstrom M, Stora J, Molnar P*, et al.* (2009) Ancient DNA reveals lack of continuity between neolithic hunter-gatherers and contemporary Scandinavians. Curr Biol 19: 1758-62. doi: 10.1016/j.cub.2009.09.017

Mathieson I, Lazaridis I, Rohland N, Mallick S, Patterson N, Roodenberg SA*, et al.* (2015) Genome-wide patterns of selection in 230 ancient Eurasians. Nature 528: 499-503. doi: 10.1038/nature16152

Nagy D, Tomory G, Csanyi B, Bogacsi-Szabo E, Czibula A, Priskin K*, et al.* (2011) Comparison of lactase persistence polymorphism in ancient and present-day Hungarian populations. Am J Phys Anthropol 145: 262-9. doi: 10.1002/ajpa.21490

Olalde I, Allentoft ME, Sanchez-Quinto F, Santpere G, Chiang CW, DeGiorgio M*, et al.* (2014) Derived immune and ancestral pigmentation alleles in a 7,000-year-old Mesolithic European. Nature 507: 225-8. doi: 10.1038/nature12960

Olalde I, Schroeder H, Sandoval-Velasco M, Vinner L, Lobon I, Ramirez O*, et al.* (2015) A Common Genetic Origin for Early Farmers from Mediterranean Cardial and Central European LBK Cultures. Mol Biol Evol 32: 3132-42. doi: 10.1093/molbev/msv181

Plantinga TS, Alonso S, Izagirre N, Hervella M, Fregel R, van der Meer JW*, et al.* (2012) Low prevalence of lactase persistence in Neolithic South-West Europe. Eur J Hum Genet 20: 778-82. doi: 10.1038/ejhg.2011.254

Ploszaj T, Jerszynska B, Jedrychowska-Danska K, Lewandowska M, Kubiak D, Grzywnowicz K, *et al.* (2015) Mitochondrial DNA genetic diversity and LCT-13910 and deltaF508 CFTR alleles typing in the medieval sample from Poland. Homo 66: 229-50. doi: 10.1016/j.jchb.2014.11.003

Sverrisdottir OO, Timpson A, Toombs J, Lecoeur C, Froguel P, Carretero JM, *et al.* (2014) Direct estimates of natural selection in Iberia indicate calcium absorption was not the only driver of lactase persistence in Europe. Mol Biol Evol 31: 975-83. doi: 10.1093/molbev/msu049

Witas HW, Ploszaj T, Jedrychowska-Danska K, Witas PJ, Maslowska A, Jerszynska B, *et al.* (2015) Hunting for the LCT-13910*T allele between the Middle Neolithic and the Middle Ages suggests its absence in dairying LBK people entering the Kuyavia region in the 8th millennium BP. PLoS One 10: e0122384. doi: 10.1371/journal.pone.0122384
